# DREADD-Induced Silencing of the Medial Olfactory Tubercle Disrupts the Preference of Female Mice for Opposite-Sex Chemosignals

**DOI:** 10.1523/ENEURO.0078-15.2015

**Published:** 2015-09-22

**Authors:** Brett T. DiBenedictis, Adaeze O. Olugbemi, Michael J. Baum, James A. Cherry

**Affiliations:** 1 Department of Biology, Boston University, Boston, Massachusetts 02215; 2 Department of Psychological and Brain Sciences, Boston University, Boston, Massachusetts 02215

**Keywords:** DREADD, hM4Di, olfactory tubercle, retrograde tract tracing

## Abstract

Attraction to opposite-sex pheromones during rodent courtship involves a pathway that includes inputs to the medial amygdala (Me) from the main and accessory olfactory bulbs, and projections from the Me to nuclei in the medial hypothalamus that control reproduction. However, the consideration of circuitry that attributes hedonic properties to opposite-sex odors has been lacking. The medial olfactory tubercle (mOT) has been implicated in the reinforcing effects of natural stimuli and drugs of abuse. We performed a tract-tracing study wherein estrous female mice that had received injections of the retrograde tracer, cholera toxin B, into the mOT were exposed to volatile odors from soiled bedding. Both the anterior Me and ventral tegmental area sent direct projections to the mOT, of which a significant subset was selectively activated (expressed Fos protein) by testes-intact male (but not female) volatile odors from soiled bedding. Next, the inhibitory DREADD (designer receptors exclusively activated by designer drugs) receptor hM_4_Di was bilaterally expressed in the mOT of female mice. Urinary preferences were then assessed after intraperitoneal injection of either saline or clozapine-N-oxide (CNO), which binds to the hM_4_Di receptor to hyperpolarize infected neurons. After receiving CNO, estrous females lost their preference for male over female urinary odors, whereas the ability to discriminate these odors remained intact. Male odor preference returned after vehicle treatment in counterbalanced tests. There were no deficits in locomotor activity or preference for food odors when subject mice received CNO injections prior to testing. The mOT appears to be a critical segment in the pheromone–reward pathway of female mice.

## Significance Statement

This work adds to a growing body of evidence that the medial olfactory tubercle (often thought of as solely an olfactory cortical structure) encodes natural, reinforcing hedonic behaviors. Females’ innate preference for male urinary odors was abolished when the mOT was silenced using DREADD (designer receptors exclusively activated by designer drugs) methodology, but persisted under control conditions wherein the medial olfactory tubercle (mOT) was not silenced. Importantly, this effect was not due to a deficit in olfactory processing (i.e., an inability to discriminate between male and female urinary odors). Moreover, the female mOT is selectively activated by male urinary odors, and this activation appears to be driven mainly by medial amygdaloid and ventral tegmental area efferents. The mOT is a key node in the pheromone–reward pathway in mice.

## Introduction

The processing of social chemosignals (or “pheromones”) in the rodent brain occurs via hardwired circuitry involving either or both the main and accessory olfactory systems. For example, Choi et al. (2005) describe a “reproductive pathway” that transmits stimuli detected by the vomeronasal organ (VNO) to the accessory olfactory bulbs (AOBs), and from there, to the medial amygdala (Me), which receives input from both the AOB and the main olfactory bulb (MOB). In turn, several nuclei in the medial hypothalamus receive projections from the Me. This model, however, does not incorporate structures known to be critical for attributing rewarding properties to opposite-sex pheromones, including regions of the ventral striatum (VS) that are required for sexual attraction (Novejarque et al., 2011; Agustín-Pavón et al., 2014; DiBenedictis et al., 2014b).

The nucleus accumbens (Acb) plays an important role in the reinforcing effects of drugs of abuse as well as natural stimuli (Roberts et al., 1977, 1980; McGregor and Roberts, 1993; Baker et al., 1998; Liao et al., 2000; Ikemoto and Sharpe, 2001; Rodd-Henricks et al., 2002; Heinz et al., 2009; Koob and Volkow 2010; Wang et al., 2013; Cassataro et al., 2014). However, additional evidence also implicates the medial olfactory tubercle (mOT) in both drug-induced as well as natural reinforcement (Ikemoto, 2003; Ikemoto et al., 2005; FitzGerald et al., 2014). The mOT is a trilaminar structure that includes the cell bridges of the ventral pallidum (VP), the islands of Calleja (ICj), as well as a striatal component, consisting mainly of GABAergic medium spiny neurons (Ikemoto, 2010). Furthermore, the mOT receives direct input from the main olfactory bulb, and recent evidence suggests that the mOT is an important center for encoding odor valence (White, 1965; Schwob and Price, 1984a; Wesson and Wilson 2011; Gadziola et al., 2015). Tract-tracing studies in female mice have shown that the mOT receives dense monosynaptic input from the Me (Pardo-Bellver et al., 2012; DiBenedictis et al., 2014a), and other results suggest that the preference of female mice to investigate opposite-sex chemosignals may involve the mOT (Agustín-Pavón et al., 2014). In that study, the medioventral striatopallidum (mvStP), a region including but not limited to the mOT, was damaged with electrolytic lesions that may have destroyed fibers of passage and also, in some cases, included adjacent brain regions. Moreover, it is unclear from that study whether the deficit in preference for male bedding odors reflects a hedonic/motivational defect or an inability to discriminate male versus female odorants. This distinction is especially important since the mvStP contains portions of the olfactory tubercle, a cortical olfactory structure. Finally, it remains to be seen whether male bedding preference deficits persist when females have access to only the volatile components of the stimulus, activating only the main olfactory system (MOS). A strong demonstration that the mOT participates in processing attractive chemosignals would involve showing that the mOT receives inputs from the “reproductive pathway” that is selectively activated by opposite-sex odors in addition to demonstrating that selective inactivation of mOT neurons eliminates attraction to opposite-sex chemosignals. We addressed these issues in two experiments. First, we used tract tracing in combination with male urinary odor-induced Fos coexpression to identify forebrain regions in female mice that innervate the mOT and are selectively activated by opposite-sex odors. Next, we used the pharmacosynthetic DREADD (designer receptors exclusively activated by designer drugs) approach to reversibly silence mOT neurons in female mice (Armbruster et al., 2007; Rogan and Roth, 2011; Farrell and Roth, 2013). Our data suggest that activity in mOT neurons plays an essential role in motivating estrous female mice to seek out male pheromones.

## Materials and Methods

### Subject mice

Seventy-four female and 12 male Swiss-Webster mice were purchased (Charles River Laboratories) at 5-6 weeks of age and were housed in same-sex groups under a reversed 12 h light/dark cycle. At Charles River Laboratories, the pregnant Swiss-Webster female was removed from the male’s cage and placed in a maternity cage well before parturition. Thus, the female offspring used in the present study did not have mating experience with a male, nor had they direct nasal access to breeding male odors prior to arriving at Boston University. Females in the functional tract-tracing study (Experiment 1) were housed four per cage for the duration of the experiment, while females in the DREADD study (Experiment 2) were housed four per cage until 48 h prior to the start of behavioral testing, whereupon they were housed individually. All behavioral testing was video recorded and conducted under red light during the dark phase of the photoperiod. Food and water were provided *ad libitum*, except where otherwise noted. The Boston University Institutional Animal Care and Use Committee approved all procedures used in this study. Three days after arrival, female subject mice as well as female bedding/urine donors underwent bilateral ovariectomy and were allowed 1 week to recover. Animals were implanted subcutaneously at the back of the neck with an estradiol (E_2_) capsule at the time of ovariectomy (bedding/urine donors and Experiment 1 subject mice) or at the time of DREADD virus or vehicle infection surgery (Experiment 2 subject mice).

### Reagents

For all surgeries, subject mice were anesthetized with 2% isoflurane vapor and were administered carprofen (5 mg/kg, s.c.) analgesic daily for 2 d after surgery. Estradiol was administered in polymeric silicone SILASTIC capsules (inner diameter, 1.57 mm; outer diameter, 2.41 mm; length, 5 mm) diluted 1:1 with cholesterol. Progesterone (P; 500 μg, s.c.) was injected 4 h prior to testing or urine collection to induce full behavioral estrus (E_2_ plus P). The retrograde tracer, cholera toxin B (CTb; List Biological Laboratories) was used at 0.5% in 0.1 m phosphate buffer, pH 6.0. In the DREADD experiment, an adeno-associated virus containing the Cre recombinase-independent viral construct AAV5-hSyn-HA-hM_4_D(Gi)-IRES-mCitrine (University of North Carolina Vector Core) was used to express the hM_4_Di receptor and the fluorescent reporter mCitrine in neurons. When bound to the nonendogenous ligand clozapine-N-oxide (CNO), hM_4_Di induces somatic hyperpolarization and markedly reduces presynaptic neurotransmitter release (Armbruster et al., 2007; Ferguson et al., 2011; Rogan and Roth, 2011; Stachniak et al., 2014). Where used, CNO (Enzo Life Sciences) was administered intraperitoneally in saline vehicle at 5 mg/kg 30 min prior to testing. This dosage and time of administration have been used previously to activate DREADD receptors (Sasaki et al., 2011; Garner et al., 2012; Farrell et al., 2013; Peñagarikano et al., 2015). Immunolabeling procedures used primary antibodies for Fos, CTb, neuronal-specific nuclear protein (NeuN), and green fluorescent protein (GFP; used to visualize mCitrine^+^ neurons; obtained from Santa Cruz Biotechnology, List Biological Laboratories, EMD Millipore, and MBL International, respectively), with secondary antibodies that included biotinylated donkey anti-rabbit (for Fos, Jackson ImmunoResearch), biotinylated donkey anti-goat (for CTb, Jackson ImmunoResearch), biotinylated horse anti-mouse/rabbit (for NeuN, Vector Laboratories), and Alexa Fluor 488 donkey anti-goat (for GFP, Life Technologies). ABC reagents, diaminobenzidine (DAB) and VectaShield mounting medium with 4',6'-diamidino-2-phenylindole dihydrochloride (DAPI) were obtained from Vector Laboratories.

### Urine and soiled bedding collection

Urine used for odor preference, odor discrimination, and terminal odor exposure was collected from group-housed, testes-intact male (*n* = 12) and E_2_-implanted ovariectomized female (*n* = 12) donor mice using metabolic cages. Pooled urine was aliquoted into 1 ml vials according to sex and stored at −20°C until use. The same donor mice were placed in a cage with clean Aspen chip bedding for 24 h. The soiled bedding was then collected and stored according to sex at −20°C until used as an olfactory stimulus for terminal odor exposures. Female bedding donors were first brought into estrus with a P injection 4 h before being placed in the cage.

### Stereotaxic injections

Seven days following bilateral ovariectomy, mice were anesthetized and the head was secured in a stereotaxic apparatus (David Kopf Instruments). Small holes were then drilled bilaterally over each injection site (coordinates: anterior–posterior, 5.3 mm from interaural line; medial–lateral, 0.7 mm from sagittal suture; depth, 4.7 mm from dura). For retrograde tracer injections (Experiment 1), a glass micropipette (30 μm tip diameter) containing CTb was lowered into the rostral mOT and deposited iontophoretically by passing a pulsatile (7 s on, 7 s off) cathodal current (+5 μA) for 5-8 min using a current source (Stoelting). The pipette was left in place for 5 min postinjection and was withdrawn from the brain under a −5 μA anodal current to prevent backflow. For Experiment 2, 0.2-0.3 μl of either the virus (hM_4_Di) or sterile PBS (vehicle) was pressure injected at 0.25-0.3 μl/min using a 5 μl syringe with a 30 gauge needle (Hamilton Company) at the stereotaxic coordinates given above. The needle tip was left in place for 5-9 min before retracting. After injection, drill holes were filled with bone wax and incisions were closed with sutures.

### Behavioral tests

#### Locomotor activity

Subject mice were given an injection of CNO or saline solution 30 min before being placed individually in Plexiglas boxes (57 × 14 × 19 cm) inside an isolation cubicle (61 × 65 × 51 cm). The movements of subject mice in the open field were tracked for the next 20 min using a digital video camera and Any-Maze software (Stoelting). Subject mice underwent two tests, one with and one without CNO, in a counterbalanced order separated by 2 d. The mice did not receive P prior to these tests. The mean distance traveled by mice in each group was compared using a two-way repeated-measures ANOVA, with infection type (hM_4_Di vs vehicle) and drug treatment (CNO vs saline solution) as factors.

#### Urinary odor preference

Two days following locomotor testing, subject mice received a noncontact, volatiles-only odor preference test for testes-intact male versus estrous female urine. Testing took place in the home cage, wherein subject mice were simultaneously presented for 5 min with the two odors (20 μl each absorbed onto filter paper) placed 7 cm apart. To restrict access to volatiles only, a wire mesh was placed over the odor source such that direct nasal contact was prevented. The location of urinary odors was switched for each test to control for any side bias. This test was administered twice (separated by 4 d), in the presence or absence of CNO in a counterbalanced order. As in other studies (Keller et al 2006; Martel and Baum, 2009b; Brock et al, 2011), subject mice that had been previously implanted with E_2_ capsules were brought into estrus with an injection of P 4 h prior to each odor preference test. These combined hormone treatments, when given to ovariectomized females, induce all aspects of feminine courtship behavior, including lordosis and the motivation to seek out male chemosignals.

Four days later, preferences for urinary odors when nasal contact was permitted were assessed. The procedures followed for these tests of volatiles plus nonvolatiles were identical to the volatiles-only tests except that the wire mesh barrier was removed, allowing direct nasal access to the urine. Again, two tests were given at 4 d intervals using a counterbalanced (with or without CNO) design with P administered 4 h prior to testing. The volatiles-only test was used to assess the specific contribution of the MOS, while the volatiles-plus-nonvolatiles test assessed the contribution of both the MOS and AOS in pheromone processing.

Time spent actively investigating (defined as having the snout raised and oriented toward and within 1 cm of the stimulus) each odor during the 5 min test was recorded. Two-way repeated-measures ANOVAs [followed by Student–Newman–Keuls (SNK) *post hoc* tests, where applicable] were then used to assess the effects of infection type and drug treatment on difference scores (time spent investigating intact male urine minus time spent investigating estrous female urine) for volatiles-only and volatiles-plus-nonvolatiles tests as well as on total investigation times (time spent investigating intact male urine plus time spent investigating estrous female urine) for each test.

#### Odor discrimination

To verify that subject mice could discriminate between testes-intact and estrous female urinary volatiles, subject mice underwent a home-cage habituation/dishabituation test. Subject mice did not receive P prior to these tests, since it has been previously shown in mice that both ovary-intact females (Isles et al., 2002) and ovariectomized females given E_2_ only (Martel and Baum 2009b; DiBenedictis et al., 2014b) can reliably discriminate between male and female urinary odors, as indexed by robust habituation/dishabituation responses to each urinary odor. Briefly, subject mice were given three presentations of water followed by three presentations of estrous female urinary odor followed by three presentations of testes-intact male urinary odor. Physical access to urine was prevented using a wire mesh barrier. Subject mice underwent two tests (separated by 2 d) in the presence or absence of CNO using a counterbalanced design. Paired *t* tests were used to compare the mean investigation times for the dishabituation responses of each group: the third presentation of water versus the first estrous female urinary odor, as well as the third presentation of estrous female urinary odor versus the first presentation of testes-intact male urinary odor. The dishabituation responses were compared between groups using a two-way repeated-measures ANOVA with infection type and drug treatment as factors.

#### Cookie odor preference

Food was removed, and subject mice were given 2.5 g of a Nutter Butter cookie. After 2 h, residual cookie crumbs were removed from the cage and subject mice were deprived of food for 24 h. A 5 min odor preference test was then administered in a format identical to the volatiles-only odor preference test, except that the odors used were 20 μl of cookie dissolved in mineral oil versus mineral oil alone. Subject mice underwent two tests (separated by 2 d) in the presence or absence of CNO in a counterbalanced order. Odor locations were switched for each test to prevent side bias. Subject mice did not receive P prior to this test. The mean times spent investigating each stimulus (cookie vs mineral oil) were calculated for each group and compared using paired *t* tests.

### Terminal odor exposure

Subject mice were habituated in an odor exposure cage (28 × 16.5 × 12.7 cm) for 4 h in a dark fume hood. Because stress has been shown to induce Fos expression in many forebrain regions (including hypothalamic and amygdaloid nuclei; Cullinan et al., 1995), we used a setup in which subject mice are not handled by the experimenter at the onset of odor exposure. This setup consisted of an exposure cage with a perforated floor that could be stacked on a second cage containing bedding that could not be physically contacted. To optimize females’ physiological responses to biological odors, Experiment 1 subject mice were given an injection of P and placed in an exposure cage that was stacked on a cage with clean bedding. After 4 h, the exposure cage was placed over a second cage containing either clean bedding or soiled bedding from testes-intact males or estrous females. Experiment 2 subject mice were treated in the same manner, except that these mice were always exposed to testes-intact male soiled bedding following a 4 h habituation. To ensure an adequate odor environment, the male-soiled bedding and the estrous female-soiled bedding were spiked with 1 ml of male or estrous female urine, respectively, and cages were placed on a heating pad on low heat (∼30°C) for the duration of exposure to maximize odor volatility.

### Histological analysis of CTb uptake and hM4Di infection

For CTb and Fos double labeling, tissues were first Fos labeled, then refixed in 4% paraformaldehyde (PFA) for 10 min and washed in PBS before staining for CTb. Briefly, 30 μm free-floating cryosections were incubated overnight in rabbit anti-c-Fos (1:1000) at room temperature followed by 1 h of incubation with biotinylated donkey anti-rabbit secondary antibody. Sections were next incubated with ABC reagent and visualized using DAB with nickel enhancement. Sections were refixed for 10 min in 4% PFA before immunolabeling for CTb, as follows: goat anti-CTb primary antibody (1:40,000) followed by biotinylated donkey anti-goat secondary antibody, incubation with ABC reagent, and visualization using DAB without nickel enhancement. Thus, two different chromogens (DAB-nickel, black nucleus; and DAB-only, brown cytoplasm) were used to identify Fos and CTb immunoreactivity, respectively. After labeling, sections were mounted, rinsed in 50% ethanol, and coverslipped. Cells that expressed hM_4_Di were identified using immunodetection with anti-GFP antibody, which also labels mCitrine (the reporter coexpressed with hM_4_Di) and quantified in epifluorescent photomicrographs (Colwill and Gräslund, 2011). Immunolabeling for mCitrine was necessary for signal detection due to low/insufficient endogenous fluorescence. These sections were placed on slides and coverslipped with VectaShield containing DAPI (1.5 μg/ml). For six subject mice in Experiment 2, additional brain sections were also immunolabeled for the neuronal marker NeuN. Counts of NeuN-labeled cells were then used in combination with adjacent DAPI sections to estimate the proportion of cells in the mOT that were neuronal.

### Specific procedures for each experiment

#### Experiment 1: identification of brain regions that project to the mOT and are activated by opposite-sex odors

**Figure 1.**
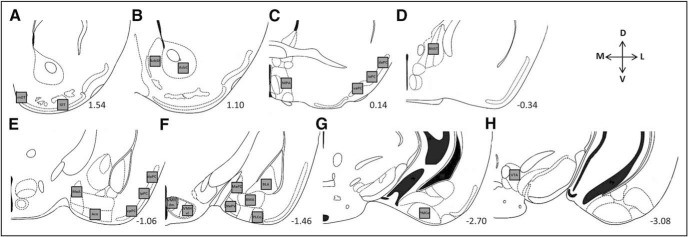
Modified schematic from the mouse brain atlas of Franklin and Paxinos (2008) showing the forebrain regions in which Fos-IR, CTb-IR, and Fos-IR^+^ CTb-IR cells (light gray boxes; 300 μm^2^) were counted. ***A–H***, Counting regions (from left to right) included the mOT and lOT (Fos only; ***A***); the AcbSh and Acb core (AcbC; ***B***); the medial preoptic area (MPA), dorsal (daPC), intermediate anterior PC (iaPC), and ventral anterior PC (vaPC; ***C***); the pBNST (***D***); the MeA, anterior cortical amygdala (Aco), dorsal PC (dpPC), intermediate PC (ipPC), and vpPC (***E***); the dorsomedial division of the ventromedial hypothalamus (VMHdm), the ventrolateral division of the ventromedial hypothalamus (VMHvl), MePD, MePV, basomedial amygdala (BMA), BLA, and PLCo (***F***); PMCo (***G***); and VTA (***H***). Numerical values represent the distance (in millimeters) from bregma for each section (Franklin and Paxinos 2008).

A pilot study involving three subject mice with unilateral injections of the retrograde tracer CTb in the rostral mOT found that there are negligible contralateral projections to the mOT. Therefore, mice were given bilateral injections of CTb into the rostral mOT with the goal of examining only the ipsilateral hemisphere with the most accurate injection. Five days after CTb injections, behavioral estrus was induced (E_2_ plus P), and mice were exposed to testes-intact male soiled bedding/urine, estrous female soiled bedding/urine, or clean bedding/water prior to being killed. Brains were removed and subsequently processed for CTb and Fos double labeling. Because Fos labeling may be compromised in neurons at injection sites, the analysis of Fos induction in rostral mOT and lateral olfactory tubercle (lOT) could be made only in hemispheres where CTb injections missed these areas. Thus, mice with inaccurate or absent CTb deposits bilaterally (*n* = 12) and/or appreciable spread of tracer into adjacent regions (*n* = 4) were excluded from the analysis of retrograde CTb labeling, but some of these mice were included in the analysis of mOT and lOT Fos expression. These missed injections were either very dorsal or lateral to the mOT, so it is unlikely that Fos induction in mOT neurons was perturbed in these cases. For brain regions outside of the injection sites, the following two different types of neuronal cell body labeling were quantified without knowledge of the treatment group: (1) mOT projecting, but not odor activated (CTb labeled only); and (2) both mOT projecting and odor activated (CTb/Fos double labeled). CTb and Fos labeling were defined based on differences in color and cellular localization (Fos: black nucleus; CTb: brown cytoplasm) using a light microscope with a 40× (oil) objective. Fos^+^ and CTb^+^ cells were counted in 21 forebrain regions in a standard (300 μm^2^) counting area using the cell counter plugin in ImageJ (Schneider et al., 2012) and averaged from two non-overlapping tissue sections for each of the brain regions for each subject (Fig. 1*A–H*). The percentage of CTb^+^ cells that coexpressed Fos was calculated from the average of two tissue sections in each of 13 forebrain regions where CTb/Fos colabeling was observed. Comparisons among the three odor exposure groups (i.e., clean, male-soiled bedding, or female-soiled bedding) were performed using separate one-way ANOVAs run for each of the 23 brain regions for Fos alone and 13 brain regions for CTb/Fos colabeling. Because the ventral tegmental area (VTA) and the anterior portion of the Me (MeA) were designated a priori as areas of interest, the α level for statistical significance of the omnibus *F* tests for these areas was set at 0.05. For ANOVAs run for all other brain regions, the α level was adjusted using the Benjamini–Hochberg correction (Benjamini and Hochberg, 1995). For ANOVAs where the omnibus *F* test result was significant, differences between odor exposure groups were subsequently examined by *post hoc* SNK tests.

#### Experiment 2: DREADD-induced silencing of mOT neurons

Ovariectomized, E_2_-treated subject mice were given bilateral injections of either sterile PBS (vehicle) or virus containing the hM_4_Di construct into the rostral mOT. Only subject mice with bilateral hM_4_Di infections centered in the rostral mOT (*n* = 7) were used for analysis along with vehicle-injected controls (*n* = 12). Subject mice with significant viral spread into adjacent nuclei bilaterally (*n* = 4) or those lacking detectable infection in one or both hemispheres (*n* = 7) were excluded from behavioral analysis. Behavioral testing commenced 3 weeks after infection. Subject mice underwent, in the following order, locomotor testing, volatiles-only odor preference testing, volatiles-plus-nonvolatiles odor preference testing, odor discrimination tests, and cookie odor preference tests. Each type of test was conducted once with and once without CNO treatment (counterbalanced). Either 4 d (for the first three test types) or 2 d (the final two test types) intervened before the next type of test. Finally, 2 d after the cookie test, subject mice were exposed to volatile male odors in either the presence or absence of CNO treatment, whereupon they were killed by perfusion 90 min after exposure onset. Brains were removed, cryosectioned, and processed for immunolabeling of Fos protein. Although mice could be tested both with and without CNO present in the behavioral studies, subject mice could receive only one or the other treatment prior to being killed for Fos analysis. Because DREADD infections in each hemisphere of a subject are distinct, Fos was analyzed in both hemispheres of each subject, and “hemisphere” was used as the unit of analysis. Fos counts within the infected rostral mOT zone were averaged from two non-overlapping sections of each hemisphere. The mean number of Fos^+^ cells in hemispheres from each group was compared using a two-way ANOVA followed by an SNK *post hoc* test.

DREADD infection rates were determined using additional sections that were immunolabeled for mCitrine and costained with DAPI. The total number of mCitrine^+^ neurons divided by the total number of DAPI^+^ cells counted in three non-overlapping sections (rostral to caudal) of the rostral mOT were computed for each subject. Because DAPI labels neuronal as well as non-neuronal nuclei, a separate estimate of neuronal infection in the mOT was made. For this analysis, three tissue sections (rostral, intermediate, and caudal mOT) that were adjacent to sections in which DAPI counts were made were identified from six subject mice in the study. These sections were immunolabeled with NeuN, and the proportion of NeuN^+^ DAPI^+^ cells in the mOT were computed for each set of adjacent sections. These values were then averaged for each subject and expressed as the mean ± SEM for the six animals.

## Results

### Experiment 1

**Figure 2.**
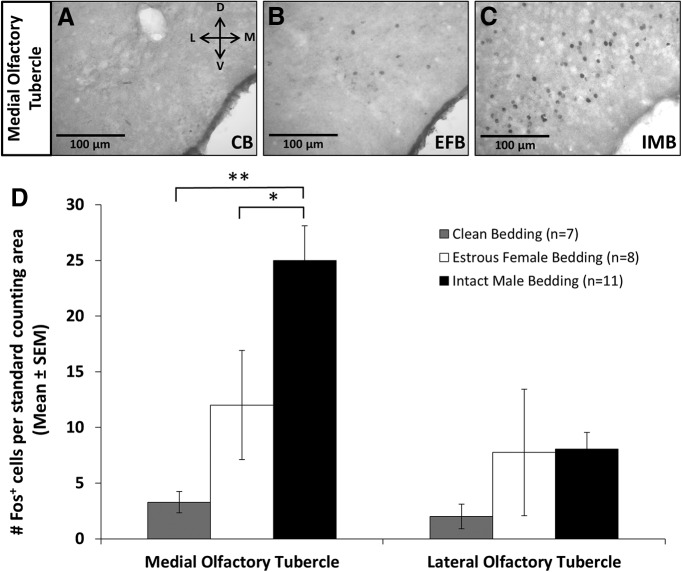
mOT neurons, but not lOT neurons, were selectively activated in female mice by volatiles emitted from opposite-sex (male) soiled bedding***. A****–****C***, Representative photomicrographs depicting Fos protein immunoreactivity in the mOT of female subject mice exposed to volatiles from clean bedding (CB; ***A***), estrous female soiled bedding (EFB; ***B***), and testes-intact male soiled bedding (IMB; ***C***). ***D***, Average number of Fos-IR cells (±SEM) observed in the mOT and lOT in response to volatiles from clean bedding, estrous female soiled bedding, or testes-intact male soiled bedding. *p < 0.01; **p < 0.001 (SNK *post hoc* tests following a significant overall ANOVA). In the legend, *n* refers to the number of subject mice in each group.

To determine whether mOT neurons are activated by volatile odors in estrous female mice, E_2_ plus P-treated subject mice were exposed to volatiles from clean bedding, estrous female bedding, or intact male bedding; and forebrains were immunostained for Fos protein (Fig. 2*A–C*). In the mOT (*F*_(2,22)_ = 9.9, *p* = 0.001^a^), but not the lOT (*F*_(2,22)_ = 1.3, *p* = 0.300^b^), male odors increased neuronal Fos induction relative to female or control odors (Fig. 2*D*). This male urinary odor-induced increase in Fos protein was not confined to a specific layer of the mOT. Rather, there was an apparent increase in Fos expression in both the dense cell layer and the multiform layer, which includes portions of the VP and the ICj. Selective Fos expression specifically to male, but not to estrous female, volatile odors was also found in the following regions: the shell of the Acb (AcbSh), VTA, MeA, the posterodorsal portion of the Me (MePD), the posteroventral portion of the Me (MePV), the posteromedial cortical amygdala (PMCo), and the posteroventral portion of piriform cortex (vpPC). A number of areas responded equally to odors from males or females, while in other regions there was no Fos induction in response to either odor compared with clean bedding (Tables 1, 2).

**Table 1:** Effect of different volatile odor stimuli (soiled bedding/urine) on forebrain Fos expression in estrous female mice

Brain region (*n*)	Volatile odor stimuli			
	Clean bedding (*n* = 8)	Estrous female bedding/urine (*n* = 8)	Intact male bedding/urine (*n* = 11)	*F*_(2,27)_ (*p* value)
Reward-associated nuclei				
AcbC	0 ± 0	0 ± 0	1 ± 0	1.6 (0.219)^c^
AcbSh*	0 ± 0	1 ± 1	11 ± 4	4.4 (0.036)^d^
VTA*	1 ± 1	3 ± 2	19 ± 6	4.9 (0.017)^e^
BLA	2 ± 1	5 ± 2	7 ± 2	1.2 (0.174)^f^
Vomeronasal recipient amygdaloid nuclei				
MeA*	2 ± 1	5 ± 2	13 ± 3	6.6 (0.005)^g^
MePD*	0 ± 0	3 ± 1	10 ± 2	7.9 (0.019)^h^
MePV*	1 ± 1	2 ± 1	11 ± 3	5.9 (0.025)^i^
PMCo*	1 ± 1	4 ± 1	9 ± 2	6.6 (0.024)^j^
pBNST	1 ± 0	2 ± 1	1 ± 1	1.1 (0.301)^k^
BMA	1 ± 1	5 ± 2	5 ± 2	1.8 (0.205)^l^
Main olfactory recipient amygdaloid and cortical nuclei				
Aco†	1 ± 0	10 ± 4	13 ± 3	4.4 (0.036)^m^
PLCo†	0 ± 0	3 ± 1	6 ± 2	4.6 (0.036)^n^
daPC	3 ± 3	11 ± 4	11 ± 4	1.3 (0.275)^o^
iaPC†	2 ± 2	9 ± 4	15 ± 3	4.6 (0.036)^p^
vaPC†	2 ± 1	7 ± 3	14 ± 3	6.1 (0.025)^q^
dpPC	3 ± 2	4 ± 2	11 ± 2	3.5 (0.064)^r^
ipPC	2 ± 1	7 ± 3	10 ± 2	3.8 (0.056)^s^
vpPC*	3 ± 1	6 ± 2	14 ± 3	5.6 (0.027)^t^
Hypothalamic nuclei				
MPA	1 ± 1	8 ± 4	11 ± 3	3.0 (0.069)^u^
VMHvl	2 ± 1	4 ± 2	8 ± 4	1.2 (0.329)^v^
VMHdm	3 ± 2	6 ± 2	12 ± 3	2.4 (0.116)^w^

Data are expressed as the mean ± SEM number of Fos-IR cells per standard counting area (300 μm^2^). AcbC, nucleus accumbens core; BMA, basomedial amygdala; Aco, anterior cortical amygdala; daPC, anterodorsal division of PC; iaPC, anterointermediate division of PC; vaPC, anteroventral division of PC; dpPC, posterodorsal division of PC; ipPC, posterointermediate division of PC; MPA, medial preoptic area; VHMvl, ventrolateral division of the ventromedial hypothalamus; VMHdm, dorsomedial division of the ventromedial hypothalamus.

* For each brain region, Fos-IR in response to intact male odor is significantly greater compared with the other two odor groups.

† For each brain region, the male odor response is significantly greater than clean bedding, but odor groups do not differ from each other [SNK *post hoc* tests following a significant (*p* < 0.05) omnibus *F* test after Benjamini–Hochberg correction for multiple testing]. The *p* values for MeA and VTA, which were areas of a priori interest, were not corrected.

**Table 2:** Summary of statistical analyses

	Data structure	Type of test	Power
a	Normally distributed	One-way ANOVA with SNK *post hoc*	0.956
b	Normally distributed	One-way ANOVA with SNK *post hoc*	0.086
c–w*	Normally distributed	One-way ANOVA with SNK *post hoc* after Benjamini–Hochberg correction	0.581 (0.062–0.900)
x	Normally distributed	One-way ANOVA with SNK *post hoc*	0.583
y	Normally distributed	One-way ANOVA with SNK *post hoc*	0.642
	Normally distributed	Two-way RM ANOVA with SNK *post hoc*	
z		Drug treatment	0.551
aa		Drug treatment × infection type	0.612
	Normally distributed	Two-way RM ANOVA with SNK *post hoc*	
bb		Drug treatment × infection type	0.415
	Normally distributed	Two-way RM ANOVA with SNK *post hoc*	
cc		Drug treatment	0.738
	Normally distributed	Two-way RM ANOVA	
dd		Drug treatment	0.050
ee		Infection type	0.050
ff		Drug treatment × infection type	0.050
	Normally distributed	Paired *t* tests, third water vs first EFU	
gg		Test group: hM4Di + saline	0.998
hh		Test group: hM4Di + CNO	0.783
Ii		Test group: vehicle + CNO	1.000
jj		Test group: vehicle + saline	0.991
	Normally distributed	Paired *t* tests, third EFU vs first IMU	
kk		Test group: hM4Di + saline	1.000
ll		Test group: hM4Di + CNO	1.000
mm		Test group: vehicle + CNO	0.999
nn		Test group: vehicle + saline	0.997
	Normally distributed	Two-way RM ANOVA	
oo		Drug treatment	0.050
pp		Infection type	0.164
qq		Drug treatment × infection type	0.052
	Normally distributed	Two-way RM ANOVA	
rr		Drug treatment	0.106
ss		Infection type	0.062
tt		Drug treatment × infection type	0.050
	Normally distributed	Paired *t* tests, cookie odor vs mineral oil	
uu		Test group: hM4Di + saline	0.800
vv		Test group: hM4Di + CNO	1.000
ww		Test group: vehicle + CNO	0.982
xx		Test group: vehicle + saline	0.988
	Normally distributed	Two-way RM ANOVA	
yy		Drug treatment	0.050
zz		Infection type	0.178
aaa		Drug treatment × infection type	0.059
	Normally distributed	Two-way ANOVA with SNK *post hoc*	
bbb		Drug treatment	0.972
ccc		Infection type	0.973
ddd		Drug treatment × infection type	0.837

Letters (left column) refer to values within the Results section. EFU, Estrous female urinary odor; IMU, testes-intact male odor; RM, repeated measures.

* Values for this series of ANOVAs are shown as the median (range) of power calculations.

**Figure 3.**
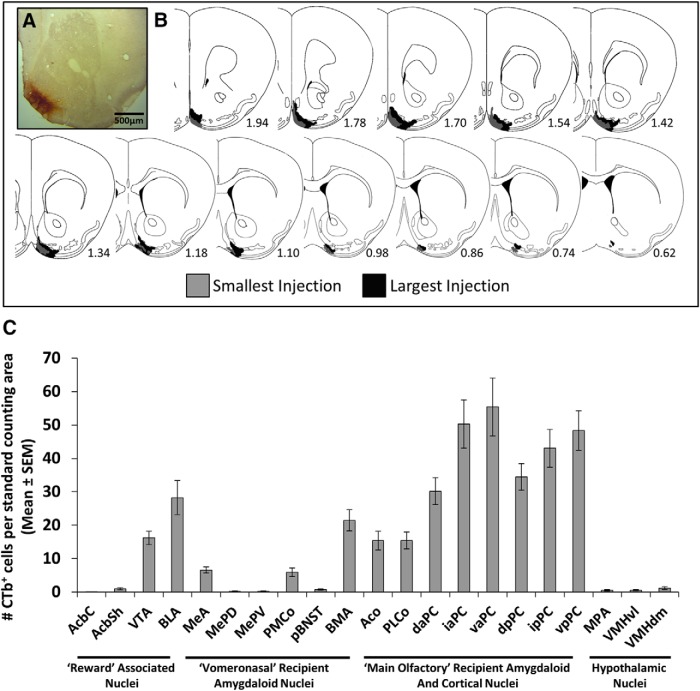
***A***, Representative photomicrograph showing a CTb injection site in the mOT. ***B***, Schematic reconstruction of coronal sections of the largest (black) and smallest (gray) injection sites in female mice given CTb injections in the mOT. Sections are ordered sequentially from anterior (left) to posterior (right), with the numbers shown representing the distance (in millimeters) anterior to bregma for each section. Adapted from Franklin and Paxinos (2008). ***C***, Mean (±SEM) number of back-labeled CTb^+^ cell bodies in various forebrain sites in female mice given CTb injections into the mOT 5 d previously (*n* = 30). AcbC, nucleus accumbens core; BMA, basomedial amygdala; Aco, anterolateral cortical amygdala; daPC, anterodosal PC; iaPC, intermediate PC; vaPC, ventral PC; dpPC, posterodorsal PC; ipPC, intermediate PC; MPA, medial preoptic area; VMHvl, ventrolateral portion of the ventromedial hypothalamus; VMHdm, dorsomedial portion of the ventromedial hypothalamus.

**Figure 4.**
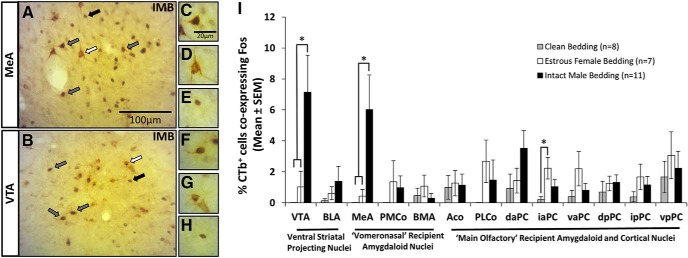
A subset of retrogradely labeled neurons in the MeA and VTA in female mice given a prior injection of CTb in the mOT coexpressed Fos in response to opposite-sex (male) volatile odors from soiled bedding (IMB). ***A***, ***B***, Representative photomicrographs depicting back-labeled CTb^+^ (brown) and Fos^+^ (black) neurons in the MeA (***A***) and the VTA (***B***). White arrows point to neurons positive for CTb; black arrows point to neurons positive for Fos; gray arrows point to neurons positive for both CTb and Fos (CTb^+^/Fos^+^). ***C****–****H***, High-magnification (100×) photomicrographs depicting colabeled (CTb^+^/Fos^+^) neurons identified by gray arrows in the MeA (***C***) and VTA (***F***), neurons positive for CTb only identified by white arrows in the MeA (***D***) and VTA (***G***), and neurons positive for Fos only identified by black arrows in the MeA (***E***) and VTA (***H***). ***I***, Effect of volatiles emitted from soiled bedding on the expression of Fos in various forebrain neurons of estrous female mice that were retrogradely labeled by a prior injection of CTb into the mOT. The mean percentage (±SEM) of CTb-labeled (mOT-projecting) cells that coexpressed Fos in response to volatiles from clean bedding, estrous female soiled bedding, or testes-intact male soiled bedding is shown in 13 forebrain regions where Fos/CTb colocalization was observed. *p ≤ 0.05 (SNK *post hoc* tests following a significant overall ANOVA). In the legend, *n* refers to the number of subject mice in each group. See the legend of Figure 3 for definitions of brain region acronyms.

The retrograde tracer CTb was used to identify brain regions that send axonal projections to the mOT. Evaluation of CTb injection sites indicated that the tracer extended from rostral to caudal regions of the mOT (Fig. 3*A*,*B*). Back-labeled CTb^+^ cell bodies were found in many ventral forebrain regions (Fig. 3*C*). The densest labeling occurred in main olfactory recipient amygdaloid and cortical nuclei. Regions of the vomeronasal amygdala and structures associated with the mesolimbic dopamine system were also labeled. To examine whether any of these mOT-projecting regions were activated by urinary volatiles, double-labeled sections (Fig. 4*C*,*F*) were analyzed to determine the extent of Fos (nucleus) and CTb (cytoplasm) colocalization. Of the 13 forebrain regions analyzed (Fig. 4*I*), exposure to male, but not female, volatile chemosignals resulted in significant CTb/Fos colabeling only in the Me and VTA (Fig. 4*A–I*; Me: *F*_(2,25)_ = 4.4, *p* = 0.023^x^; VTA: *F*_(2,25)_ = 4.9, *p* = 0.017^y^).

### Experiment 2

#### Behavior

**Figure 5.**
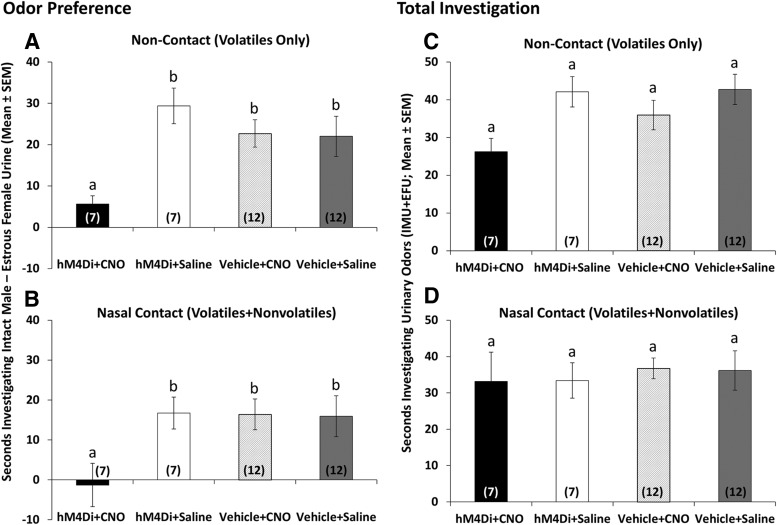
***A***, ***B***, Effect of bilateral CNO-induced mOT silencing (hM_4_Di+CNO) on the preference of ovariectomized, estradiol- and progesterone-primed female mice to investigate urinary odors from estrous female vs testes-intact male mice presented simultaneously in the home cages of subject mice. ***A***, ***B***, The preference of subject mice for volatile urinary odors presented outside the home cage (Non-Contact – Volatiles Only; ***A***) and the preference of subject mice for volatile plus nonvolatile urinary odors presented inside the home cage (Nasal Contact − Volatiles+Nonvolatiles; ***B***). Data are represented as the average (±SEM) time spent investigating intact male urine minus the time spent investigating estrous female urine for each group. Different letters above the columns in each group indicate statistically significant differences from each other (two-way repeated-measures ANOVA with one-factor repetition followed by SNK *post hoc* test). The number of subject mice in each group is given within columns in parentheses. ***C***, ***D***, Effect of bilateral CNO-induced mOT silencing (hM_4_Di+CNO) on the total amount of time ovariectomized, estradiol-primed, and progesterone-primed female mice spent investigating urinary stimuli. ***C***, Total amount of time subject mice spent investigating intact male plus estrous female urinary volatiles (Non-Contact − Volatiles Only). ***D***, Total amount of time subject mice spent investigating intact male plus estrous female urinary volatiles and non-volatiles (Nasal Contact − Volatiles plus Nonvolatiles). Data are represented as the average (±SEM) time spent investigating intact male urine plus the average time spent investigating estrous female urine for each group (*p* > 0.05, two-way repeated-measures ANOVA with one-factor repetition). The number of subject mice in each group is given within columns in parentheses.

DREADD-induced silencing of the mOT was examined for effects on olfactory behavior. Preference for testes-intact male versus estrous female urinary volatiles was abolished in hM_4_Di-plus-CNO subject mice (*p* = 0.012), whereas preference for male chemosignals was maintained in hM_4_Di plus saline, vehicle plus CNO, and vehicle plus saline subject mice [Fig. 5*A*; main effect of drug treatment (*F*_(1,37)_ = 6.0; *p* = 0.026^z^) and drug treatment × infection type interaction (*F*_(1,37)_ = 6.7; *p* = 0.019^aa^) on odor investigation difference scores]. Similar effects were observed when direct nasal contact with the urinary stimulus was permitted (Fig. 5*B*), such that preferences were observed for testes-intact male over estrous female urine in the hM_4_Di plus saline, vehicle plus CNO, and vehicle plus saline groups, but not in the hM_4_Di plus CNO females [infection type × drug treatment interaction (*F*_(1,37)_ = 4.5; *p* = 0.048^bb^), *p* < 0.02, SNK *post hoc* tests]. The total time spent investigating urinary volatiles depended on drug treatment (Fig. 5*C*; *F*_(1,37)_ = 8.4; *p* = 0.01^cc^), an effect that appears to be driven mostly by reduced investigation of male chemosignals by hM_4_Di plus CNO subject mice. No group differences in total investigation times were observed when direct nasal access to the urinary stimulus was permitted (Fig. 5*D*; drug treatment, *p* = 0.969^dd^; infection type, *p* = 0.589^ee^; interaction, *p* = 0.934^ff^).

**Figure 6.**
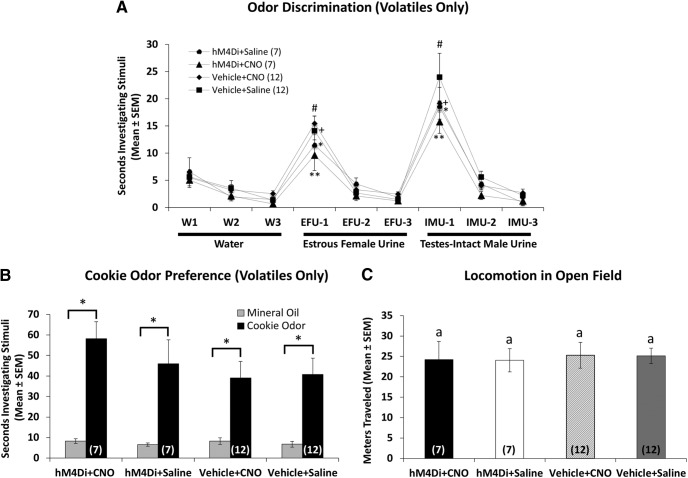
***A***, Effect of bilateral CNO-induced mOT silencing (hM_4_Di+CNO) on the ability of ovariectomized, estradiol-primed female mice to discriminate between testes-intact male and estrous female volatile urinary odors presented outside of the home cage (volatiles only). Each stimulus was presented three consecutive times. Estrous female versus testes-intact male urinary volatiles were reliably discriminated by all groups (paired *t* test comparisons of mean investigation times of third water versus first female urine, and third female urine versus first male urine; *p < 0.001; +*p* < 0.001; #*p* < 0.001; **p < 0.001). No between-group differences in the level of investigation during the first presentation of estrous female urine and the first presentation of intact male urine were observed (*p* > 0.05, two-way repeated-measures ANOVA with one-factor repetition). ***B***, Effect of bilateral CNO-induced mOT silencing (hM_4_Di+CNO) on the preference of ovariectomized, estradiol-primed female mice to investigate volatiles emanating from cookie odor dissolved in mineral oil vs mineral oil alone presented simultaneously in the home cages of subject mice. Data are represented as the mean (±SEM) time spent investigating each odor (*p < 0.01, paired *t* test comparisons of mean investigation times for each odor). ***C***, Effect of bilateral CNO-induced mOT silencing (hM_4_Di+CNO) on locomotion displayed by ovariectomized, estradiol-primed female mice. Data are represented as the mean (±SEM) distance traveled (in meters) in a 20 min open field test (*p* > 0.05, two-way repeated-measures ANOVA with one-factor repetition). The number of subject mice in each group is given within columns in parentheses.

In the odor discrimination task that was administered to ensure that DREADD-induced neuronal silencing in the mOT had no effect on the ability of subject mice to discriminate among the odors tested, all groups were dishabituated from the final presentation of water to the first presentation of estrous female urine, as well as from the final presentation of estrous female urine to the first presentation of testes-intact male urine (Fig 6*A*; all *p* ≤ 0.014^gg–nn^). Similarly, there were no group differences in the amount of time spent investigating either the first presentation of estrous female urine (main effects, *p* ≥ 0.16^oo,pp^; interaction, *p* = 0.323^qq^) or the first presentation of testes-intact male urine (main effects, *p* ≥ 0.225^rr,ss^; interaction, *p* = 0.750^tt^). All groups also strongly preferred to investigate cookie odor volatiles versus mineral oil vehicle (*p* ≤ 0.013^uu–xx^), suggesting that reduced motivation to investigate male urinary odors in hM_4_Di plus CNO subject mice is not generalized to food odors (Fig. 6*B*). Finally, no significant differences were found between groups in the mean distance traveled in a locomotor test (Fig. 6*C*; main effects, *p* ≥ 0.146^yy,zz^; interaction, *p* = 0.364^aaa^), indicating that neither CNO nor hM_4_Di receptor expression affected the motor function of subject mice.

#### Localization of hM4Di infection

**Figure 7.**
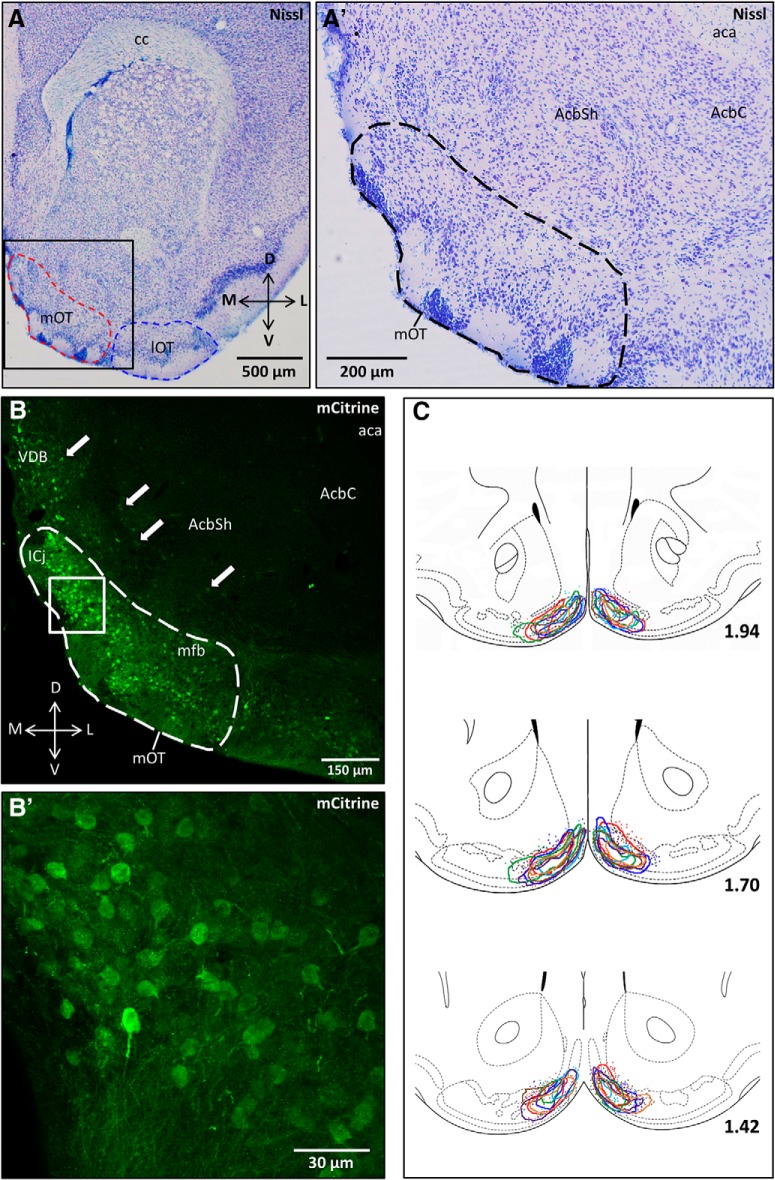
***A***, Low-magnification (4×) Nissl-stained photomicrograph of the brain tissue section containing the medial ventral striatum depicted in ***A'***, outlined by the boxed region. The red dashed region outlines the mOT, and the blue dashed region outlines the lOT. ***A'***, Nissl-stained photomicrograph depicting the medial portion of the ventral striatum (medial nucleus accumbens and medial olfactory tubercle) in an adjacent section of forebrain depicted in ***B***. The dashed region outlines the mOT. ***B***, Epifluorescent photomicrograph depicting hM_4_Di infection in the rostral mOT, immunolabeled for the coexpressed reporter mCitrine. The dashed region outlines the mOT. White arrows point to errant infected cell bodies found outside of the mOT, illustrated as colored dots outside of the traced regions in ***C***. ***B'***, z-Plane stacked confocal image showing mCitrine^+^ cell bodies and fibers magnified from the white, boxed region in ***B***. ***C***, Modified schematic from the mouse brain atlas of Franklin and Paxinos (2008) illustrating regions where hM_4_Di^+^ and DAPI^+^ cells were quantified. Different color tracings indicate the extent of bilateral hM_4_Di infection within three rostral sections of the ventral striatum for each subject (*n* = 7). Different color dots represent sparse, errant hM_4_Di^+^ neurons found outside of the densely infected (traced) region for each subject. Sections are ordered sequentially from anterior (top) to posterior (bottom), with numerical values representing the distance (in millimeters) anterior to bregma for each section. aca, Anterior commissure; AcbC, Acb core; cc, corpus callosum; mfb, medial forebrain bundle.

A typical site where mOT infections were seen is shown for a representative subject in a Nissl-stained section (Fig. 7*A*,*A'*). The expression of the DREADD construct in the rostral mOT of an adjacent section from this animal is shown using low-magnification epifluorescent (Fig. 7*B*) and high-magnification confocal microscopy (Fig. 7*B'*) after immunolabeling for mCitrine. Figure 7*C* depicts the boundaries traced around mCitrine^+^ neurons in three rostral mOT sections from each hemisphere of all subject mice (*n* = 7). Sparse, errant mCitrine^+^ cell bodies found outside of the border of the primary mOT infection are also shown. The majority of infected neurons were located in the rostral mOT, although infections often spread to include more caudal regions of the mOT. Minor bilateral viral spread outside of the mOT was in some cases observed along the border of the lateral ventricles, in the navicular postolfactory nucleus, the AcbSh, in the caudal ventral tenia tecta and in the nucleus of the vertical limb of the diagonal band of Broca (VDB). Significant unilateral spread from the caudal mOT into the VDB and/or the AcbSh was observed in three subject mice, but these animals were retained in the study since these areas were unaffected in the contralateral hemisphere. Few mCitrine^+^ cell bodies were observed elsewhere in the brain, as adeno-associated viruses are transported predominantly in the anterograde direction (Harris et al., 2012). The only region outside of the targeted infection area where mCitrine^+^ cell bodies were regularly found was in PC, although infection levels were very low (∼5-15 mCitrine^+^ cell bodies/section). mCitrine^+^ fibers were observed in many regions known to receive input from the mOT, including the MOB, anterior olfactory nucleus, posterolateral cortical amygdala (PLCo), anterior and posterior PC, and the VP, with sparse labeling observed in the caudate putamen (CPu) and VTA.

#### Estimate of hM4Di infection rates

The proportion of DAPI^+^ cells that coexpressed mCitrine was determined for the infected regions traced in the three sections shown for each subject in Figure 7*C*. These measures for each section were averaged within and across subject mice to obtain a mean ± SEM hM_4_Di infection rate of 20 ± 2%. From a separate analysis of NeuN and DAPI labeling in sections from six subject mice, it was determined that 78 ± 7.5% of DAPI-labeled cells in the mOT are neurons; accordingly, the proportion of the mOT neuronal population infected by hM_4_Di is likely >20%.

#### Odor-induced Fos

**Figure 8.**
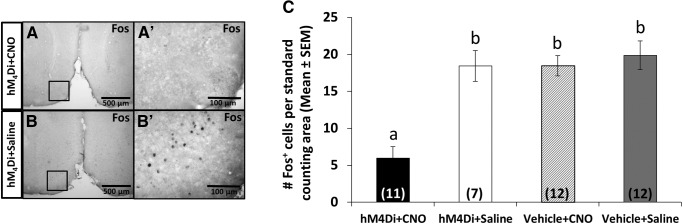
***A***, Photomicrograph depicting low Fos protein expression in response to male bedding volatiles in the mOT of a CNO-treated subject. ***A'***, High-magnification photomicrograph of the boxed area shown in ***A***. ***B***, Photomicrograph depicting augmented Fos protein expression in response to male bedding volatiles in the mOT of a saline-treated subject. ***B'***, High-magnification photomicrograph of the boxed area in ***B*** showing Fos^+^ cell bodies. ***C***, hM_4_Di+CNO-treated subject showed reduced Fos protein expression following exposure to testes-intact male soiled bedding volatiles compared with hM_4_Di+Saline treated, Vehicle+CNO-treated and Vehicle+Saline-treated subject mice. ***C***, Mean number of Fos-IR cells (±SEM) observed in the mOT in response to volatiles from testes intact male soiled bedding. Different letters above columns in each group indicate statistically significant differences from each other (two-way repeated-measures ANOVA followed by SNK *post hoc* test). The number of hemispheres examined for each group is given within columns in parentheses.

To confirm the efficacy of DREADD inhibition *in vivo*, female subject mice were exposed to volatile chemosignals from testes-intact males prior to being killed (Fig. 8*A*,*B'*). In hM_4_Di plus CNO subject mice, there was a significant reduction in mOT Fos expression compared with all other groups (Fig. 8*C*), suggesting that neuronal activity is diminished by CNO-induced DREADD receptor activation, and not by CNO alone or DREADD infection alone [main effects of drug treatment [*F*_(1,41)_ = 15.4; *p* < 0.001^bbb^) and infection type (*F*_(1,41)_ = 15.5; *p* < 0.001^ccc^), and interaction of infection type × drug treatment (*F*_(1,41)_ = 9.7; *p* = 0.003^ddd^)]. Importantly, decreased Fos expression has been used previously to confirm CNO-induced neuronal silencing in hM_4_Di-infected brain regions (Ferguson et al., 2011; Sasaki et al., 2011; Michaelides et al., 2013; Pei et al., 2014).

## Discussion

The present results indicate that neuronal activity in the mOT plays an essential role in motivating female mice to investigate male odors, providing evidence that the mOT is part of a circuit that regulates the innate attraction of mice for urinary odors. When the rostral mOT of hM_4_Di-infected estrous females was silenced with CNO, preference for intact male over estrous female urinary volatiles and nonvolatiles was abolished, whereas preference persisted when subject mice were treated with saline solution. Furthermore, CNO treatment had no effect on vehicle-injected subject mice. These findings, along with previous findings (Agustín-Pavón et al., 2014; DiBenedictis et al., 2014b), highlight the importance of the VS in the “hardwired” circuitry that underlies behavioral preferences for opposite-sex pheromones—a first step in mate recognition leading to successful reproduction.

The mOT has been linked to drug reinforcement (Ikemoto, 2010) as well as to olfactory perception (Payton et al., 2012). Thus, it is possible that the decrements we observed in the investigation of urinary odors during CNO-induced mOT inhibition were due to sensory deficits in odor detection, or to interference with the processing of odors from the direct projections of the MOB to the mOT. However, mOT-hM_4_Di^+^ subject mice treated with CNO were able to perceive differences among the urinary odors tested, as indexed by a robust dishabituation response to the first presentation of each odor. This result suggests that reduced preference for male odors did not result from females’ inability to discriminate male and female urinary odors. Instead, the mOT may modulate urinary odor-driven behaviors by attributing salience to these odors, either locally or via its reward-associated projection targets. This latter view is supported by the findings of a recent report (Gadziola et al., 2015) that the activity of OT neurons increased upon presentation of various nonpheromonal odors that predicted the delivery of a water reward to thirsty mice. In the present study, CNO-treated subject mice retained their preference for volatile cookie odors over mineral oil vehicle, indicating that the mOT may not influence the motivation of females to investigate food odors. Finally, all subject mice displayed equivalent levels of locomotor activity, regardless of whether or not they received CNO. This rules out any possibility that deficits in preferences for male odors were due to the physical inability of subject mice to approach/investigate odor stimuli.

Volatile components of odors are detected by the main olfactory system. In this pathway, sensory neurons in the main olfactory epithelium send axonal projections to the MOB, which in turn radiates information via mitral/tufted cell projection neurons to downstream targets, including olfactory cortical structures and portions of the amygdala referred to as the “olfactory amygdala” as well as nuclei of the “vomeronasal amygdala,” and particularly the medial amygdala (Kang et al., 2009). Lesions of the main olfactory epithelium eliminate male urinary volatile-induced Fos expression in olfactory targets of the female mouse forebrain (Martel and Baum, 2007), and reduce lordosis behavior and attraction male pheromones (Keller et al., 2006). These findings suggest that the main olfactory system plays an essential role in processing male pheromonal odors in female mice. In the present study, we also found a selective activation of the mOT (i.e., increased Fos expression) of females in response to volatiles from male-soiled bedding, but not from female-soiled bedding, implicating the mOT in the circuitry that processes innately attractive, opposite-sex odors. Notably, the AcbSh which receives dopaminergic inputs from the VTA, also responded preferentially to opposite-sex odors.” In our study, a small number of hM_4_Di-infected cell bodies was detected unilaterally in the ventromedial AcbSh of three subject mice; this could have contributed to the deficit in male odor preference displayed by subject mice with bilateral hM_4_Di infections of the mOT after CNO treatment. Although the AcbSh receives only sparse direct inputs from the Me (Pardo-Bellver et al., 2012; DiBenedictis et al., 2014a), it is strongly interconnected with the mOT. Thus, the AcbSh may also be involved in aspects of urinary odor-driven sociosexual motivation. More work is needed to test this hypothesis.

Deficits in the investigation of opposite-sex odors during DREADD-induced inhibition of mOT neuronal activity occurred not only in tests in which only urinary volatiles were available, but also in tests during which both volatile and nonvolatile urinary chemosignals could be detected. Nonvolatile chemosignals are processed by the accessory olfactory system (Luo and Katz, 2004), and, indeed, surgical removal of the VNO reduced the investigation of opposite-sex urinary odors in female mice (Martel and Baum 2009b). Thus, our present results show that chemosignals in testes-intact male urine, whether processed by the main and/or accessory olfactory systems, require input to the mOT to render them attractive to females.

An initial indication that the mOT may play an essential role in interpreting the salience of pheromonal cues in female mice came from a report (Agustín-Pavón et al., 2014) that bilateral electrolytic lesions of the mvStP, but not of the posterolateral striatopallidum, eliminated the preference of females to investigate male versus female chemosignals. Our results focus attention on the mOT, a subdivision of the larger mvStP, as the critical site in mediating the rewarding effects of opposite-sex pheromones, just as the mOT, as opposed to the lateral part of the OT, has been implicated in drug reward (Ikemoto, 2010). Our results using DREADD methodology show for the first time that silencing activity in the mOT eliminated the preference of females to investigate male pheromones without compromising their ability to discriminate between these odors or reducing the motivation of females to investigate food odors.

We found that a subset of neurons projecting to the mOT from the MeA and VTA showed preferential induction of Fos in response to male bedding odors compared with female bedding odors. This suggests that the MeA and VTA are key regions driving the selective activation of the rostral mOT during exposure to volatile chemosignals from testes-intact males. In corroboration, it was previously found that the MeA densely innervates the mOT (Pardo-Bellver et al., 2012; DiBenedictis et al., 2014a). Both anterior and posterior segments of the Me may also drive activity in the mOT via indirect polysynaptic inputs involving the posterior bed of the nucleus of the stria terminalis (pBNST) and PMCo (Ubeda-Bañon et al., 2008; Novejarque et al., 2011). The VTA provides dopaminergic innervation to the ventral striatum, including the Acb, VP, and OT complex, and neurons in the VTA express Fos in response to opposite-sex chemosignals (Moncho-Bogani et al., 2005; Kang et al., 2009; Martel and Baum 2009a), thereby implicating this region in the processing of salient olfactory information. It has also been shown that dopaminergic modulation of the medial Acb and mOT is necessary for the display of male urinary odor-driven courtship behaviors in estrous female mice (DiBenedictis et al., 2014b), further implicating the VTA in pheromone reinforcement. The mOT is a component of the VS, and receives pheromonal input from limbic/amygdaloid structures. Thus, our results are consistent with the hypothesis that neuronal activity in the mOT modulates urinary odor-driven motivated behaviors in mice.

DREADD methodology offers several useful advantages for studying olfactory behaviors. It is reversible, so (to our knowledge) there is no damage to temporarily silenced neurons, and it enables animals to be tested repeatedly and in alternating control and mOT-inhibited conditions. Moreover, infected neurons are easily identified and quantified using a coexpressed reporter gene. The level of viral infection in our study was sufficient to produce both functional deficits in odor-induced activation of Fos as well as in the investigation of opposite-sex chemosignals. Additionally, we calculated infection rates based on a DAPI counterstain—which labels nuclear DNA in both neurons and glia (Kapuscinski, 1995)—so it is likely that the proportion of neurons infected in the present study was higher than the DAPI-based estimate of ∼20%. In a separate examination that we conducted using brain sections that were labeled with DAPI and the neuron-specific marker NeuN, ∼78% of the total number of DAPI-labeled cells in the mOT were found to be neurons. An estimate of the proportion of neurons infected by the DREADD virus may therefore be closer to 25%.

Retrograde labeling of cell bodies in subject mice given CTb injections was found in many forebrain regions known to innervate the mOT (Newman and Winans, 1980). We found that the majority of inputs to the mOT derived from “main olfactory” recipient cortical nuclei, such as the PC, although a fair number of back-labeled cell bodies were also observed in the VTA and basolateral amygdala (BLA). Vomeronasal recipient nuclei, including the MeA and PMCo, also targeted the mOT, though to a much lesser extent than the PC. These results are consistent with those of other studies suggesting that the mOT is interconnected with olfactory structures that include the MOB, PC, and the vomeronasal amygdala, as well as hypothalamic, hippocampal, and reward-associated brain regions such as the Acb, lateral septum, VTA, VP, and CPu, among others (Wesson and Wilson, 2011). Future studies should exploit genetically guided, cell-specific techniques to activate or inhibit particular mOT neuronal populations in behaving animals in order to further specify the role of this region in mediating the effects of pheromones on courtship behaviors.

## Synthesis

The decision was a result of the Reviewing Editor Cheryl Sisk and the peer reviewers coming together and discussing their recommendations until a consensus was reached. A fact-based synthesis statement explaining their decision and outlining what is needed to prepare a revision is listed below. The following reviewers agreed to reveal their identity: Zuoxin Wang

Dear Jim, Your manuscript has been reviewed by two experts in the field and by me. We all think the research is of interest and that using DREADD to uncover a role for the mOT attraction to opposite-sex odors is a compelling approach. The reviewers have noted several instances where clarification of methods is needed or where you should consider alternative interpretations, limitations, or caveats to your conclusions. I invite you to prepare a revised manuscript that addresses the reviewers' concerns, which are provided below. Thank you for submitting your work to eNeuro.

Finally, the discussion seems to be too long and redundant, and should be shortened.

Synthesis Statement for Author (Required):

Response: We have cut some parts of the Discussion, but some text was also added to address reviewer comments. Overall, the length has been reduced from 1536 to 1473 words. We note that the Discussion was and remains well below the word limit of 3000.
